# Negative and positive consequences of adolescent cancer 10 years after diagnosis: an interview-based longitudinal study in Sweden

**DOI:** 10.1002/pon.3549

**Published:** 2014-04-15

**Authors:** Vicky Lehmann, Helena Grönqvist, Gunn Engvall, Malin Ander, Marrit A Tuinman, Mariët Hagedoorn, Robbert Sanderman, Elisabet Mattsson, Louise von Essen

**Affiliations:** 1 Department of Health Sciences, Health Psychology Research Section, University of Groningen, University Medical Center Groningen (UMCG)Groningen, The Netherlands; 2 Psychosocial Oncology and Supportive Care, Department of Public Health and Caring Sciences, Uppsala UniversityUppsala, Sweden; 3 Department of Women's and Children's Health, Uppsala UniversityUppsala, Sweden

**Keywords:** cancer, oncology, survivors, adolescence, consequences, longitudinal

## Abstract

**Objective:**

The aim of this study was to provide insight into survivor-reported negative and positive consequences of cancer during adolescence 10 years after diagnosis and compare these with consequences reported 3 and 4 years after diagnosis.

**Methods:**

Three, 4, and 10 years after diagnosis, survivors of adolescent cancer were interviewed about negative and positive consequences due to their cancer experience. Manifest content analysis was used to identify categories of reported consequences. Categories of consequences 10 years after diagnosis were compared with consequences reported 3 and 4 years after diagnosis.

**Results:**

Seven categories of negative consequences were identified: *bodily concerns*, *existential thoughts about loss and life* (new at 10 years), *psychological problems*, *difficulties interacting with others*, *health worries* (new), *fertility concerns* (new), and *frustrations about health care* (new); and six categories of positive consequences: *positive view of life*, *positive view of self*, *compassion for others* (new), *close relationships*, *gained knowledge about disease and health care*, and *financial gains*. Consistent with previous time points, *bodily concerns* were reported most often. The majority of survivors (*n* = 22) reported both negative and positive consequences of their former disease. Few reported only negative (*n* = 2) or only positive consequences (*n* = 4).

**Conclusions:**

Ten years after diagnosis, most survivors reported both negative and positive consequences. New themes, relevant to young adulthood and long-term survival, were identified. Health care professionals treating young adult survivors may anticipate and address problems regarding physical health, fertility, and health care but may also reinforce positive affect by addressing survivors' positive views of life, sense of self, and close relationships.

## Introduction

Adolescence is a crucial phase in life involving physical as well as emotional change and maturation. It seems obvious that a cancer diagnosis during this time can impact a person's life in many ways; however, our empirical knowledge about this impact is limited. Findings from previous research on potential late effects of childhood or adolescent cancer are diverse and reviews have pointed out that aggregating information, in order to pinpoint universal late effects, is complicated due to methodological issues and heterogeneous samples 1,2. Overall, differences between healthy peers and survivors concerning, for example, well-being or quality of life (using quantitative instruments) were found. However, the magnitude of differences was minor 1–3 implying that long-term survivors are doing as well as healthy controls. However, Quinn and colleagues 4 pointed out that generic health-related quality of life instruments miss several aspects that are relevant for the lives of young adults who have survived cancer during childhood or adolescence. They identified three topics that survivors missed in generic questionnaires: sense of self, relationships, and parenthood. Hence, an interview-based approach enables researchers to pinpoint aspects that are important to young adults who have survived a disease such as cancer by giving them the freedom to address aspects relevant to them and omit irrelevant issues.

Previous qualitative findings, from the present longitudinal study 5,6 and other studies 7–9 showed that survivors experience both negative and positive consequences due to their former disease. Negative consequences were reported with respect to limitations, such as physical problems, being outside the circle of friends, or having difficulties with school and work 5–9. Positive consequences, on the other hand, often concerned emotions, beliefs, and attitudes, such as a more positive view of life, personal growth, or good self-esteem 5–8.

However, qualitative studies are scarce, often cross-sectional, and include heterogeneous samples of survivors (e.g., diagnosed at age 0–18 years). Therefore, the present study was set up longitudinally to follow a group of survivors who were diagnosed during adolescence (aged 13–19 years). A longitudinal design enables the comparison of potential changes in perceived consequences of cancer over time. This is of relevance, because consequences may change during prolonged survival but may also change due to changing life phases. In general, adolescents experience not only physical changes, such as the development of secondary sex characteristics, growth, and hormonal changes, they also mature cognitively and emotionally, show increased negative affect, and often have their first sexual experiences 10. Overall, adolescence is described as a rather distressing life phase where a diagnosis such as cancer may even have a greater impact than during any other phase in life. In addition, the survivors were emerging into adulthood, a phase marked by demographic changes and unpredictability such as residential changes, career choices, and further exploration of intimate relationships 11.

Therefore, the purpose of this study was to identify survivor-reported negative and positive consequences 10 years after a cancer diagnosis during adolescence. These were compared with reported consequences at 3 and 4 years after diagnosis, in order to examine whether new themes emerged and others disappeared. In this way, we sought to identify topics specifically relevant to adolescence versus young adulthood and acute versus long-term survival.

## Method

### Data collection and measures

Throughout this longitudinal study, participants were interviewed via telephone at 4–8 weeks after diagnosis (T1); 6, 12, and 18 months (T2, T3, T4); and 2, 3, 4, and 10 years after diagnosis (T5, T6, T7, T8). Each time, survivors were asked to participate, irrespective of whether they participated at the previous time point. T8 data were collected between 2010 and 2013 and include answers to two open-ended questions: *What*, *if anything*, *is negative for you due to the cancer disease*? and *What*, *if anything*, *is positive for you due to the cancer disease*? The interviewer (GE) asked follow-up questions for clarification only, that is, did not prompt or suggest topics. The two questions were also asked 3 and 4 years after diagnosis (T6 and T7), and findings were previously published 5. The answers of participants who completed T6, T7, and T8 were used for comparisons over time.

### Sample

This longitudinal study started with 61 adolescents diagnosed with cancer who were treated in the university hospitals in Lund, Umeå, or Uppsala in Sweden (see also 5,6,12 for more detailed information) and was approved by all local ethical committees. After 10 years, 19 participants (31%) had died, and others withdrew for unknown reasons (*n* = 7) or could not be traced (*n* = 7). This resulted in a sample of 28 survivors participating 10 years after diagnosis (response rate: 67%, after excluding deceased individuals). Survivors were 23–29 years old (*m* = 25.5, *SD* = 1.5), and both genders were almost equally represented with 15 male and 13 female participants (54% vs. 46%). Diagnoses included lymphoma (*n* = 12; 43%), leukemia (*n* = 8; 29%), osteosarcoma (*n* = 4; 14%), and others (*n* = 4; 14%). Two survivors had experienced relapses within the previous 10 years, but all participants were disease-free and off treatment by the time of the current data collection.

### Data analyses

The interviews were transcribed verbatim and analyzed using manifest content analyses: recording units (i.e., words and sentences) were identified representing the manifest content of the interviews (i.e., what was said), which were grouped into categories based on their content 13. Two authors (GE and EM) used the Swedish transcripts to identify such recording units, which were translated into English. Based on the Swedish original recording units and English translations, a multi-lingual group of four authors (VL, HG, GE, and EM) grouped all recording units into mutually exclusive categories. If any identified content corresponded to categories identified at earlier measurements (T6 and/or T7, see 5), the content was given the same category label. However, we did not analyze the interviews with the purpose to fit findings into earlier identified categories. Finally, reported consequences were compared with those identified at T6 and T7.

## Results

The content of the 28 survivors' interviews 10 years after diagnosis was categorized into 13 categories of which seven include negative consequences and six comprise positive consequences (Table 1). The majority of survivors (*n* = 22) reported both negative and positive consequences. Four survivors reported only positive and another two only negative consequences. The content of all categories is described in the succeeding text and illustrated by examples.

**Table:** Categories of negative and positive consequences reported 10, 4, and 3 years after diagnosis

Category	T8 (10 years) *n*	T7 (4 years) *n*	T6 (3 years) *n*	Changes at T8
Negative consequences				
Bodily concerns	21	17	16	
Existential thoughts about loss and life	13	—	—	New
Psychological problems	9	—	—	Focus shifted
Overlap: negative self-esteem (T6/T7)	—	0	3	
Overlap: unpleasant thoughts and feelings (T6/T7)	—	6	10	
Difficulties interacting with others	8	—	—	
Health worries	8	—	—	New
Fertility concerns	4	—	—	New
Frustrations about health care	3	—	—	New
Outside circle of friends (T6/T7)	—	7	4	Disappeared
Difficulties with school/ work (T6/T7)	—	1	1	Disappeared
Time consumption and financial issues (T6/T7)	—	0	4	Disappeared
Positive consequences				
Positive view of life	19	11	15	
Positive view of self	18	—	—	Focus shifted
Overlap with: Good self-esteem (T6/T7)	—	10	13	
Compassion for others	12	—	—	New
Close relationships	10	7	12	
Gained knowledge about disease and health care	7	11	8	
Financial gains	2	3	4	
Broader perspectives (T6/T7)	—	1	1	Disappeared
*n*=	28	25*	25*	

* Participants who completed T6, T7, T8.

### Negative consequences

#### Bodily concerns

This category is characterized by physical late effects and limitations as well as appearance concerns. Numerous survivors (*n* = 21) reported physical problems that also led to limitations in daily life. For example, a worse overall physical condition, reduced heart–lung capacity, less strength, pain, an impaired immune system, or stomach and digestive problems. Limitations were reported concerning sport and leisure activities, household tasks, or being careful about what to eat. One survivor mentioned vaginal dryness as negatively interfering with her sex life (female, 25 years). Physical aspects were also reported causing negative feelings about one's appearance. Thirteen survivors (twelve of whom also reported physical limitations) described physical aspects as interfering with their looks, such as having thin hair, ugly scars, or stretch marks. Some mentioned weight gain as limiting their daily life, but also as causing negative feelings about their looks.

#### Existential thoughts about loss and life

Survivors expressed regrets and sorrows about having missed certain things in life due to their previous cancer diagnosis; such as not finishing school (male, 24 years) or declining to see a psychologist which, in hindsight, they should have done (male, 23 years). A few survivors (*n* = 3) stated that they limit themselves or feel limited due to their former disease. Some reported thoughts such as ‘what if’ [they hadn't had cancer], but acknowledged at the same time, that they do not know whether they would be better off or not. Several survivors (*n* = 8) expressed existential thoughts about life, for example, being more aware that life could end anytime, not daring to look ahead in time, or that ‘one thinks that it can never happen to you, but now you know that it can’ (female, 26 years).

#### Psychological problems

Several survivors (*n* = 9) reported feeling inferior or negative about themselves and expressed psychological and emotional struggles, such as feelings of depression and uselessness. Some said ‘I cannot say the word cancer without getting the shivers’ (male, 23 years) or labeled the former diagnosis as a ‘pest’ (male, 25 years). One survivor did put it into a time perspective by saying that during treatment she was strong, but afterwards she started feeling bad. She pointed out that attention should be paid to potential needs for psychological treatment *after* the time of treatment (female, 24 years).

#### Difficulties interacting with others

Eight survivors expressed problems and concerns interacting with others when it comes to talking about cancer. Most of them did not want to disclose their former disease to everyone and some invented stories (e.g., about car accidents) in order to avoid talking about cancer. One survivor mentioned having encountered people who thought cancer was contagious. Others did not want to deal with being labeled and stigmatized as ‘the one who has had cancer’ (female, 24 years).

#### Health worries

This category comprised two major aspects: survivors' worries about their own health and about other people's health in their environment (*n* = 6). Survivors were afraid of relapses, increased risks for new tumors due to treatment, or other late effects. They also described themselves as very attentive to the symptoms of others and one survivor made her family go to regular medical checkups.

#### Fertility concerns

A few survivors (*n* = 4) expressed concerns about infertility/sterility. One man knew about his sterility while three women were concerned about their potential infertility due to amenorrhea. These concerns negatively influenced family planning and dating; and were also labeled ‘deal-breaker’ (male, 24 years).

#### Frustrations about health care

This category includes problems with follow-up care, medication, and oncologists. Survivors (*n* = 3) expressed frustrations about long waiting times and their oncologists' behaviors. They felt that they were taken seriously at the time of cancer treatment, while currently their late effects were less urgent for their specialists. Follow-ups and the treatment of late effects were perceived as a great burden in terms of time and financial investment (which could interfere with working hours). Additionally, taking medication potentially for the rest of their lives was experienced as burdensome.

### Positive consequences

#### Positive view of life

Numerous survivors (*n* = 19) reported appreciating life greatly and not taking things for granted. They reported being less concerned with minor or unimportant things and live more in the moment since the time of their cancer diagnosis. One survivor reported sometimes being ‘unnaturally happy’ about small things such as the sun shining (female, 25 years). The survivors reported being more relaxed and to put things into perspective, because ‘it could be worse’ (male, 24 years). Several survivors expressed having adopted a perspective of ‘you never know’ when life might end and that one, therefore, should prioritize and enjoy the important things in life. Some explicitly made a comparison to their peers saying that they were wasting their time, e.g., by going to pubs (female, 24 years).

#### Positive view of self

In line with a more positive view of life, numerous survivors (*n* = 18) reported viewing themselves positively. They used descriptions such as being stronger, more mature, more secure, more driven, or less superficial after having had cancer. Some expressed feeling empowered after having overcome such a disease.

#### Compassion for others

Several survivors (*n* = 12) reported feeling empathic toward other people. They explained that by knowing what it means to be sick, they empathize with them and, at the same time, try to help them. Helping others was expressed as evoking positive feelings about themselves.

#### Close relationships

This category is characterized by numerous statements about how relationships with friends and family have become closer. Survivors (*n* = 10) reported being more open and able to discuss things within the family and that everyone cares deeply about one another. Survivors reported appreciating being with friends and family and paying specific attention to spending much time with them.

#### Gained knowledge about disease and health care

Survivors (*n* = 7) reported increased and ‘valuable’ knowledge, which they had gained due to their cancer experience. They reported having learned a lot about the body, cancer, and how hospitals work.

#### Financial gain

Two survivors reported financial advantages due to their cancer disease, by getting insurance money (male, 25 years) or claiming that the cancer experience was good for the career (female, 24 years).

### Changes over time – old and new consequences

Twenty-five of the 28 survivors in this study completed the interviews 3 and 4 years after diagnosis, and these data were used for comparisons. At all three time points, most survivors (*n* = 15/25) reported both negative and positive consequences. For some, the valence of their reported consequences changed. For example from reporting both types of consequences to reporting only positive consequences at T8 (*n* = 3) or vice versa (*n* = 2; see Table 2).

**Table 2:** Consequences reported 10, 4, and  years after diagnosis and patterns of change

	T8 (10 years)	T7 (4 years)	T6 (3 years)
Negative and positive consequences	n = 19	n = 20	n = 20
Positive consequences only	n = 4	n = 1	n = 2
Negative consequences only	n = 2	n = 3	n = 2
No consequences	—	n = 1	n = 1
Patterns of change T6-T7-T8			
Reported both types of consequences any time	n = 15		
From both types of consequences to only positive at T8	n = 3		
From only negative to both types of consequences at T8	n = 2		
From only positive to both types of consequences at T8	n = 2		
From both types of consequences to only negative at T8	n = 1		
Reported negative consequences at any time	n = 1		
From no consequences to only positive consequences at T8	n = 1		

N = 25 (completed T6, T7, T8)

The categories identified 10 years after diagnosis differed to some extent from those identified 3 and 4 years after diagnosis (Table 1). Overall, *bodily concerns* were the most frequently reported consequence, which survivors still experienced 10 years later. Survivors mentioned similar aspects at all time points, for example, pain, physical limitations, and appearance aspects. They did not report new physical aspects related to long-term survival, such as cardiac and pulmonary diseases 14,15.

However, four new negative and one new positive category of consequences were identified: *existential thoughts about loss and life*, *health worries*, *fertility concerns*, *frustrations about health care*, and *compassion for others* (described in the previous section). At the same time, four consequences from previous measurements were not mentioned anymore: *outside circle of friends* (i.e., feeling isolated or having lost touch with friends), *difficulties with school*/*work* (i.e., it costs too much effort and energy; missing a lot), *time consumption and financial issues* (i.e., treatment costs a lot of time, missing study grants), and *broader perspectives* (i.e., new leisure activities and occupational plans 5).

### Changes over time – shifted categories

In addition to identifying different categories over time, the specific content of certain categories seems to have shifted as well. Some categories were not entirely new 10 years after diagnosis but contained previously mentioned aspects with a different emphasis.

First, the reported psychological and emotional struggles were put into a broader perspective: 3 and 4 years after diagnosis, *negative self-esteem* as well as *unpleasant thoughts and feelings* were two independent categories. Ten years after diagnosis, both categories were subsumed under *psychological problems*, because some survivors still reported a negative self-esteem and unpleasant thoughts, but they were more prone to report general feelings of depression, inferiority, and uselessness, such as ‘I never feel great and satisfied’ (male, 25 years) or ‘I missed an important part of my upbringing and personal development’ (male, 25 years). Moreover, at T6 and T7, *unpleasant thoughts* were specifically reported in relation to negative memories of the disease and treatment, which was not the case at T8.

Second, while survivors named *good self-esteem* as a consequence of cancer 3 and 4 years after diagnosis, this view seems to have broadened to a more general *positive view of self* at T8, with survivors describing themselves as more mature, strong, driven, confident, or as a good person.

Other categories such as *positive view of life*, *close relationships*, *gained knowledge about disease and health care*, and *financial gains* are comparable across all measurement points.

## Discussion

In this article, we present survivor-reported negative and positive consequences of cancer during adolescence, 10 years after diagnosis. Because of the longitudinal design of this study, these were compared with consequences reported 3 and 4 years after diagnosis. Most survivors reported both negative and positive consequences at all time points. However, 10 years after diagnosis, as the survivors reached emerging or young adulthood, new categories were identified, previously identified categories disappeared 5, and certain foci shifted within categories.

The most commonly reported negative consequences were *bodily concerns* at 3, 4, and 10 years after diagnosis. These problems were similar across time and no new physical issues arose during prolonged survival. Besides causing limitations, survivors described their physical problems as interfering with their appearance, which is in line with findings from other research 7,16.

However, because survivors had reached another phase in life, that is, emerging/young adulthood, new concerns emerged related to their previous disease. First, *thoughts about loss and life* concerned sorrows and regrets about having missed opportunities, which can be closely tied to emerging/young adulthood where career and study choices are vital 11. Second, concerns about (potential) infertility were experienced as negative and hindering romantic relationships. Previous research has addressed infertility/sterility in a medical sense (e.g., 17,18), and some studies have identified long-term childhood cancer survivors' worries about fertility 7,16,19,20. However, only a few studies have addressed how infertility can negatively influence romantic relationships in the context of cancer 20–24. Third, survivors described new problems in the health care system due to their extended survival, giving them the feeling that their problems were less urgent. Note that, in Sweden, survivors receive follow-up care at their regional pediatric oncology centers until age 18 years. Afterwards, they are transferred to adult oncology/hematology units. Overall, the newly identified categories concerning existential thoughts, (in)fertility, and frustrations about health care can be understood in relation to the new life phase survivors approached and their extended time of survival. In addition, *health worries* (about the own and other people's health) may not be an overall new topic that survivors have not been concerned with before, but it may be more central due to their maturation. At the same time, previously reported themes disappeared possibly due to the new life phase: being outside the circle of friends and problems with school may be prominent in adolescence but less significant as survivors aged and emerged into young adulthood 11.

Positive consequences, reported 10 years after diagnosis, concerned affect and emotions related to how survivors appreciate life, their sense of self, and the closeness they experience with family and friends. Generally, these consequences were in line with those reported at previous measurements of this longitudinal study 5,6 and with findings from others 7,25. However, several survivors emphasized the joy and gratitude they gain from helping others, which was not mentioned at earlier time points. Overall, this may be conceptualized as posttraumatic growth (PTG), that is, the experience of a positive psychological change resulting from a struggle with challenging or traumatic circumstances 26. Recently, posttraumatic growth has also been commonly identified in a group of adolescent and young adult survivors of childhood cancer 27. However, the development of compassion and empathy for others may also be linked to the general maturation and development from adolescence to emerging/young adulthood 11,28.

Overall, the negative and positive categories identified in this study are in line with previous research, but go beyond. For example, a recent meta-synthesis including 17 qualitative studies 29 on adolescent and young adult survivors shortly after diagnosis identified topics such as physical functioning, importance of support, striving for normality, impact of diagnosis, and positive experiences such as re-evaluating life. The present study adds valuable knowledge to the current literature by identifying new themes relevant to long-term survival and young adulthood, such as fertility concerns and problems with health care, but also positive aspects such as compassion for others; topics usually not identified shortly after diagnosis.

Although the majority of survivors reported both negative and positive consequences related to their former disease, a few struggled to accept and integrate the fact that they have had cancer in their life stories. One survivor referred to cancer as a ‘pest’ and some avoided talking about it. Two survivors only identified negative consequences related to their former disease. The notion that a few survivors struggle in the long-run is supported by a recent large-scale quantitative study 30. The authors followed childhood cancer survivors for more than a decade and identified four classes of survivors' well-being based on levels of depression, anxiety, and somatization. Survivors were either performing on a good level, low level, with increasing, or with decreasing levels of symptoms over time. Survivors who were doing well made up more than two-thirds of the sample and those who were getting worse, in terms of higher depression or anxiety made up only a small proportion of the sample (10–12%), which could be seen as comparable with our findings. However, we were unable to identify any attributes that these survivors shared in our sample, such as cancer type, age, gender, or having had a relapse.

This study provides important indications about how adolescent cancer influences survival in the long-run, but some limitations need to be addressed. First, the sample size is rather small, but a quite homogenous group in terms of age has been recruited, and the fact that 67% of them were followed over a period of one decade is a strength of this study. Second, with open-ended questions respondents may forg**e**t to mention certain aspects or deliberately chose to avoid talking about certain topics. This may imply an under-reporting of consequences. Third, we did not examine the perceived severity of the reported consequences, which should be incorporated in future research.

To conclude, the main findings of this study are the following: physical problems were the most common problem for survivors of adolescent cancer, even after a period of 10 years. Additionally, new topics relevant to the current life phase (i.e., emerging and young adulthood) such as fertility and family planning, and some relevant to long-term survivorship, such as problems with the health care system, were identified. At the same time, numerous positive cognitions and attitudes were expressed. Health care professionals should anticipate and address the identified problems relevant to life phase and long-term survival, but may also reinforce positive affect by addressing the survivors' positive views of life, sense of self, and close relationships. Future research should address and quantify the identified consequences in larger samples of cancer survivors, and examine whether certain consequences might be more relevant for specific types of cancer, treatment modalities, gender, or different time points during survival.

## Conflict of interest

The authors have declared no conflicts of interest.
